# Division of the Muscles of the Eye for Strabismus

**Published:** 1841-01

**Authors:** J. H. Dix

**Affiliations:** Boston


### *Division of the Muscles of the Eye for Strabismus.* By J. H. Dix, M. D., Boston.

*Division of Internal Rectus. October* 16, 1840. Miss Mary M. C., æt. twenty-three, of Boston, has squinted from birth with both eyes, the left being more decidedly turned in, the edge of the cornea of which frequently reaches the inner canthus. She can on closing the right, turn the left out nearly as far as the other, but cannot keep it fixed there. Vision has always been weak, and she is conscious that this eye does not assist in seeing.

Drs. Reynolds, Jeffries, Hooper, Bethune, and Charles Ware, were present. The eyes being small and the patient very timid, I found it necessary to control the globe by fixing a double hook through the conjunctiva into the tunica albuginea about a line and a half from the edge of the cornea towards the inner canthus. By this means the eye being fairly everted, a fine, sharp hook was passed through the conjunctiva, and an incision made about half way between it and the double hook. A blunt hook was then brought under the muscle, and a division made with scissors in the muscle about half an inch from the cornea, which Mr. Guthrie, whose large experience renders his authority decisive, says is preferable to a division of the tendon. On opening both eyes after the trifling hæmorrhage had ceased, the left eye is observed to be straight, the squint of the right being as before. Apply compress with cold water. Keep both eyes covered.

*17th.* No pain, but complains that the eye feels heavy. Bandage removed.

*22d.* There is considerable fungous growth from the place of the incision, which is cut off with scissors.

*30th.* Miss C. has been working at her trade as a tailoress for several days past, and says that after the day's work she finds the eye somewhat turned in. In the morning it is straight. The fungous growth has re-appeared, and requires to be touched with nitras argenti.

*Nov.* 18. Since the last date Miss C. has been obliged to apply herself sedulously to sewing, and the eye is turned in somewhat, a little more perhaps than the right, with which, as was at first stated, she squints a little. Still the eye is by no means so much turned in as before the operation; she has the privilege, as she terms it, of turning it out when she pleases, and finds it serviceable to vision, until it is fatigued by long use. She proposed to have the muscle re-divided when she has an opportunity for resting the eye for a time. The fungous growth has at length disappeared, and the redness at the inner canthus diminished.

*Division of Internal Rectus.—Oct.* 16. Mrs. I., æt. twenty-six, of Boston, squinted with right eye at nine months of age, immediately after hooping-cough. The squint is very decided, a portion of the cornea being hidden at the inner canthus. Drs. Doane, Dale, and Parkman, being present, the operation was performed as in the preceding case, except that the patient possessing considerable fortitude, I dispensed with the use of the speculum and the double hook, with which in the first case the eye was turned out. She kept the eye steadily everted a little towards the outer canthus. On removing the instruments the eye was found to be perperfectly straight, and capable of turning inward very little. Compress wet with cold water to be constantly kept on the eye, both being covered at the same time. Light diet.

*18th.* Mrs. I. suffering no pain, and having had none except for about eighteen hours after the operation, and then not severe, I directed the eyes to be uncovered.

*20th.* Has had some pain, about as much as immediately after the operation, which may perhaps be attributed to her getting chilled by exposure to the night air while looking from the window at a fire. Renew application of cold water. Sulph. magnesia ℥i.

*21st.* Eye comfortable. At several times since the operation she has had double vision for an hour at a time, but she has not observed it for three days past. Vision is much clearer and stronger than before the operation, and the only difference perceptible in the eyes, which are both perfectly straight, is that the right cannot be turned to the inner canthus quite so far as the left.

*Nov.* 22. Eyes perfectly straight, and motions of both parallel, the right having a farther movement inward than immediately after the operation.—*Med. Examiner for Nov.*

